# Neuroscience of cancer: Research progress and emerging of the field

**DOI:** 10.1002/ibra.12172

**Published:** 2024-08-14

**Authors:** Issam AbuQeis, Yu Zou, Ying‐Chun Ba, Abeer A. Teeti

**Affiliations:** ^1^ Department of Radiology Palestinian Ministry of Health Ramallah Palestine; ^2^ Department of Anatomy, Institute of Neuroscience, School of Basic Medicine Kunming Medical University Kunming China; ^3^ Department of Chemistry, School of Science Hebron University Hebron Palestine; ^4^ Department of Epidemiology, School of Public Health Kunming Medical University Kunming China

**Keywords:** cancer invasion, neuroscience, paracrine mode, perineural invasion, tumor microenvironment

## Abstract

Cancer cells immediately expand and penetrate adjoining tissues, as opposed to metastasis, that is the spread of cancer cells through the circulatory or lymphatic systems to more distant places via the invasion process. We found that a lack of studies discussed tumor development with the nervous system, by the aspects of cancer‐tissue invasion (biological) and chemical modulation of growth that cascades by releasing neural‐related factors from the nerve endings via chemical substances known as neurotransmitters. In this review, we aimed to carefully demonstrate and describe the cancer invasion and interaction with the nervous system, as well as reveal the research progress and the emerging neuroscience of cancer. An initial set of 160 references underwent systematic review and summarization. Through a meticulous screening process, these data were refined, ultimately leading to the inclusion of 98 studies that adhered to predetermined criteria. The outcomes show that one formidable challenge in the realm of cancer lies in its intrinsic heterogeneity and remarkable capacity for rapid adaptation. Despite advancements in genomics and precision medicine, there is still a need to identify new molecular targets. Considering cancer within its molecular and cellular environment, including neural components, is crucial for addressing this challenge. In conclusion, this review provides good referential data for direct, indirect, biological, and chemical interaction for nerve tissue–tumor interaction, suggesting the establishment of new therapy techniques and mechanisms by controlling and modifying neuron networks that supply signals to tumors.

## Graphical abstract

Cancer cells immediately expand into the neighboring normal tissues by invasion process, which is the spread of cancer cells through the circulatory or lymphatic systems to more distant places, and the neuron–cancer interaction and functional effect could basically be surmised by the influence of the nervous system elements on the normal cells and in parallel to given cancer cells, although nervous system promotes tumor metastasis through biological effects by perineural invasion and chemical effects by modulation of metastatic cascades via releasing of neural‐related factors from nerve endings of chemical substances such as neurotransmitters and neurotropic factors by a mechanism called a paracrine mode.

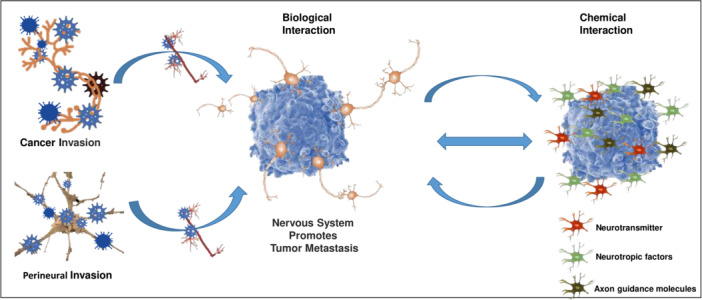

## 1 BACKGROUND

The nervous system has a wide range of appreciating functions in our bodies either in healthy or pathological situations, by maintaining homeostasis and regulating organogenesis, and usually described as signaling structures, the function of which is primarily associated with chemical and electrical transmission.^1^ Nerves are essential for the body's internal communication and proper physiological regulation by connecting organs to the central nervous system (CNS), in parallel it takes a substantial role in oncology growth and regulation, as well as progression, regeneration, and immune functions. Over the last few years, researchers have increasingly been curious about the new field of cancer neuroscience,^2^ sparking the research on the mechanisms of nervous system–cancer interactions. These mechanisms include electrochemical interactions, paracrine interactions from neurons/nerves to cancer cells, systemic nervous system–cancer interactions, and interactions between “neurons or nerves, cancer cells, and immune cells.” In summary, cancer cells may influence cell‐intrinsic signaling and other processes classically associated with neural cells, and cancer therapies can profoundly alter nervous system function.^3^

The emerging importance of nerves in cancer progression reflects on the recently reported nerve dependence in tissue regeneration. The molecular mechanisms of nerve dependence remain to be fully explored.^4^ In the regenerative blastema and the tumor, there is a bidirectional communication between growing nerves and proliferating cells,^5^ and the nerve–stem cell interaction is an area that deserves further investigation. There is proven evidence that axonogenesis is stimulated by malignant cells and gives a share in cancer growth and metastasis.^6^ Till now, there are no samples that could indicate how targeting nerves may impact cancer growth in patients and surgical denervation.^7^

Cancers are categorized according to two main criteria: (1) histological type, based on the tissue of origin, and (2) primary site, which refers to the location in the body where the cancer initially develops.^8^ Histologically, there exist numerous cancer types, which are typically grouped into six major categories including carcinoma, sarcoma, myeloma, leukemia, lymphomas, and mixed types.^9^ Carcinoma denotes a malignant neoplasm originating from epithelial cells, affecting either internal or external body linings. These malignancies, representing 80%–90% of all cancer cases, primarily involve epithelial tissue.^10^ Sarcoma refers to cancer that arises in supportive and connective tissues, including bones, tendons, cartilage, muscles, and fat. It predominantly occurs in young adults, with the most common type often manifesting as a painful bone mass. Sarcoma tumors typically exhibit characteristics resembling the tissue in which they originate,^11^ whereas myeloma develops in the plasma cells located in the bone marrow, responsible for producing certain blood proteins.^12^ The fourth category leukemia, which originates in the bone marrow, is characterized by the overproduction of immature white blood cells. It encompasses various types such as myelogenous, lymphoblastic, polycythemia vera, and others.^13^ Lymphomas originate in the glands or nodes of the lymphatic system, which includes vessels, nodes,^14^ and organs such as the spleen,^15^ tonsils,^16^ and thymus.^17^ This system purifies bodily fluids and generates infection‐fighting white blood cells or lymphocytes. Therefore, the last category is called mixed types, which are components that may be within one category or from different categories such as adenosquamous carcinoma,^18^ mixed mesodermal tumor,^19^ carcinosarcoma,^20^ and teratocarcinoma.^21^ However, a good example of a tumor that affects the nervous system is the glioma.

Glioma, the predominant type of neoplasm in the CNS, represents the nerve–cancer interaction^22^ because it originates from the abnormal proliferation of glial cells, which normally provide support to nerves and contribute to the functioning of the CNS.^23^ Predominantly found in the brain but also capable of forming in the spinal cord, gliomas are characterized by their malignant nature, signifying their cancerous attributes. Despite some gliomas exhibiting a gradual growth rate, they remain primary brain tumors, exclusive to the brain tissue. Noteworthy is the limited propensity for gliomas to metastasize beyond the confines of the brain or spinal cord.^24^ Nevertheless, their inherent life‐threatening nature arises due to the challenges posed in surgical interventions, with the tumors often infiltrating intricate regions of the brain. The multifaceted treatment of gliomas typically involves a combination of surgical procedures, radiation therapy, and chemotherapy, the selection of which is contingent upon factors such as tumor type, grade, location, and the overall health status of the afflicted individual. The prognosis for gliomas varies considerably, underscoring the imperative for ongoing research to advance therapeutic modalities for these formidable CNS neoplasms.^25^

High‐grade gliomas stand as the primary contributor to cancer‐related mortality within the CNS for both pediatric and adult populations. The persistent clinical challenges associated with these tumors underscore the inadequacy of the current comprehension of glioma pathophysiology. Though gliomas exhibit extensive infiltration within the brain and spinal cord, occurrences of growth beyond the confines of the CNS are exceptionally uncommon.^26^ The progression of gliomas is not solely governed by cell‐intrinsic mechanisms; instead, it is influenced significantly by critical microenvironmental dependencies. Notably, neurons emerge as a pivotal constituent of the glioma microenvironment, exerting a regulatory role in malignant growth through an activity‐dependent mechanism.^27^ High‐grade gliomas are categorized based on their level of aggressiveness, determined by the rate of growth, into either anaplastic astrocytomas (grade III) or glioblastoma multiform (GBM). These tumors frequently infiltrate the neighboring healthy tissue, posing a significant challenge for surgical removal.^28^ Therefore, given the heterogeneous microenvironment and the extensive infiltration of tumors within CNS, it is necessary to elucidate the cancer invasion from the perspective of neuroscience.

This study carefully demonstrates and describes the cancer invasion in the nervous system, and all aspects of its invasion such as chemical interaction, biological interaction, direct/indirect interactions, and their effect mechanisms. In addition, it reveals the research progress and emerging neuroscience of cancer. These findings contribute to a deeper understanding of interactions between cancer and the nervous system, potentially guiding future therapeutic strategies.

## 2 METHODOLOGY AND REVIEW PROCESS

**Figure 1:**
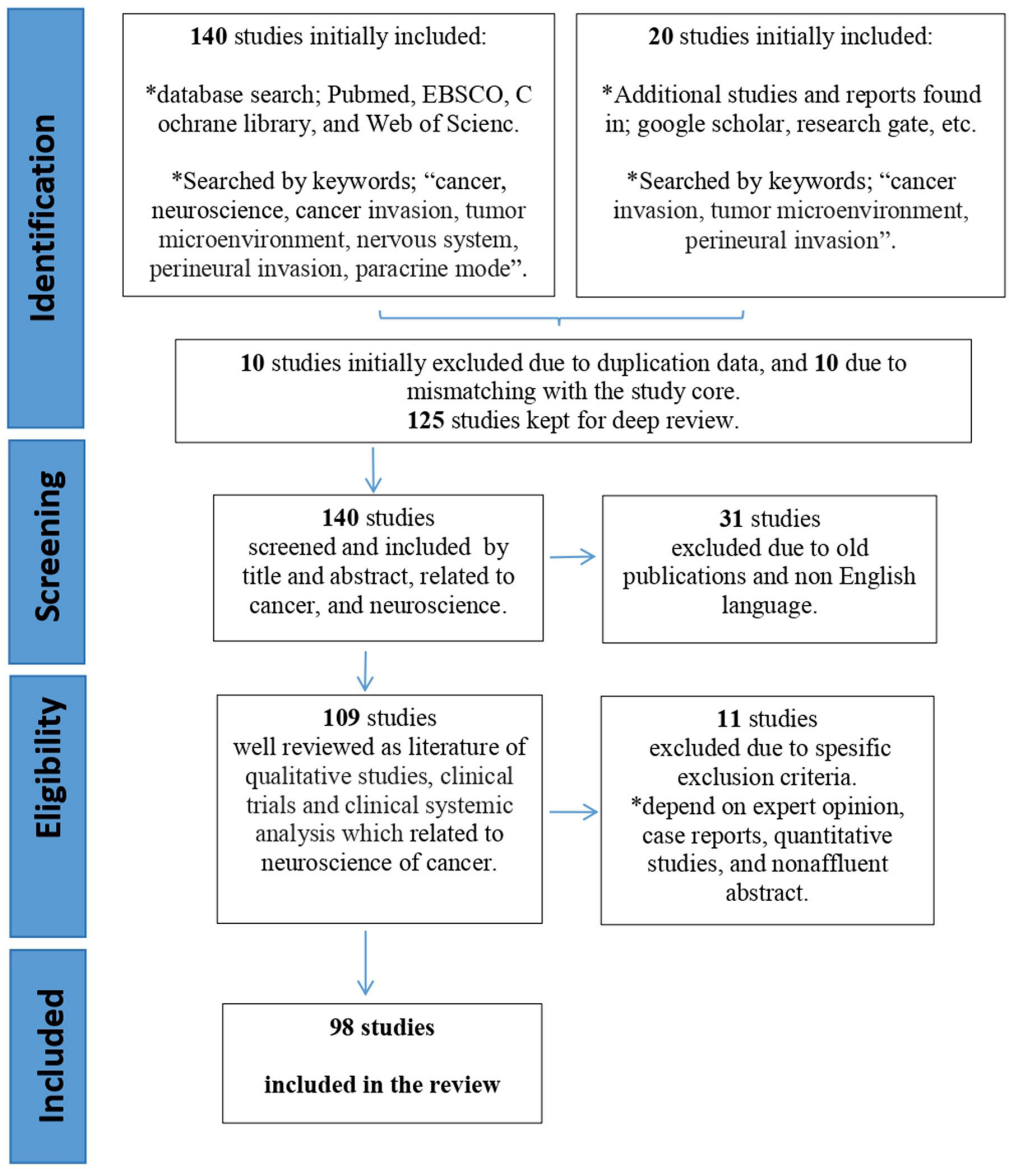
PRISMA and schematic flowchart of inclusion/exclusion criteria. PRISMA, preferred reporting items for systematic reviews and meta‐analyses. [Color figure can be viewed at wileyonlinelibrary.com]

The methodology employed in this study relied on the examination and summarization of 160 studies. Subsequently, the main outcomes of these studies were subjected to analysis. The primary objective was to comprehend the relationship between the nervous system and cancer growth, specifically investigating how cancer invades neurons. Furthermore, the study delved into the chemical and biological mechanisms underlying this invasive process. The inclusion and exclusion criteria are shown in Table 1, whereas in Figure 1, the preferred reporting items for systematic reviews and meta‐analyses (PRISMA) flowchart describes the schematic flow of inclusion and exclusion criteria we have employed and the minimized data to achieve the study's eligibility.

**Table 1:** Inclusion/exclusion criteria.

Index	Inclusion criteria	Exclusion criteria
Types	Prospective/retrospective cohort, systemic review, meta‐analysis, randomized controlled trial, etc.	*Case reports, experts' opinions, and personal websites
		*Nonaffluent abstract (lack of required data)
Time of the study	New publications after 2008–2023	Old publications before 2008
Language of the study	Publications in English or translated to English	Non‐English publications
Methods	*Qualitative	*Quantitative
	*Studies depend on clinical trials and clinical systemic analysis	*Studies depend on questionnaire or interview

## 3 LITERATURE ANALYSIS AND FINDINGS

### 3.1 Cancer—Neuronal connection

The neuron–cancer interaction and functional effect could basically be surmised by the influence of nervous system elements on the normal cells and in parallel to a given cancer cell.^29^, ^30^ In the CNS, glutamatergic neuronal activity promotes and boosts glial precursor cell proliferation,^30^, ^31^ as well as the growth of malignant gliomas, and through bona fide neuron‐to‐glioma synapses, the malignant cells can electrically merge into neural circuitry. Thus, through the glioma cell membrane, potential depolarization of the postsynaptic electrical signaling would promote cancer progression.^32^

Cancers also influence nervous system function by affecting the neural signals that connect the brain with other parts of the body, leading to remodeling and dysfunction of the nervous system. Released signals from brain tumors, such as gliomas impact the function of invaded neural circuits by encouraging aberrant synaptogenesis, increasing neuronal excitability, and causing seizures,^33^ then the neuronal activity will be increased by this pathological mechanism and in turn would promote the activity‐dependent signals that induce glioma growth.^32^ In the peripheral nervous system (PNS), in the tumor microenvironment cancers induce axonal ingrowth, where nerve density is strongly associated with cancer aggressiveness in many tumor types.^34^ In addition, malignancy cells invade the peripheral nerve tissue by a mechanism called “per neural invasion” and result in the remodeling of these peripheral nerves and chronic pain syndromes.^35^ Here, we explored neuron–cancer interaction from the chemical and biological interaction.

#### 3.1.1 Chemical connection

Amino acids such as neuropeptides (NPs) contribute to various physiological and homeostatic processes in both the CNS and PNS. Besides, it secretes other neurotransmitters such as gamma‐aminobutyric acid (GABA) and glutamate. Neuropeptide Y (NPY) is secreted from tumor cells and acts through multiple receptors, especially the Y2 receptor (Y2R). It mediates proliferation and angiogenesis during cancer development.^36^, ^37^ Therefore, the correlation between NPs and malignancy cell prognosis is scientifically evident. Several NPs have been identified that play a significant role in stimulating various important signaling pathways, thus leading to colorectal cancer (CRC) development.^38^ Additional studies have been done with immunohistochemical analysis of the NPY system in prostate pathology, demonstrating that NPY is involved in many biological mechanisms, including the regulation of cell growth and survival. Morphological analysis has shown a homogeneous membrane and cytoplasmic pattern of NPY staining in cancer cells and its membrane localization with apical accentuation in benign prostate (BP) glands.^39^

The intricate interplay between neuronal activity and cancer progression, particularly within the CNS and PNS, is a subject of profound interest. Notably, evidence from Barron et al. suggests that inhibiting the β2‐adrenergic signaling pathway, potentially through the use of beta‐blockers, may exert a mitigating effect on breast cancer progression and mortality.^40^ However, the mechanistic underpinnings of how nerves precisely contribute to cancer progression remain ambiguous. The role of Schwann cells in this context is highlighted, demonstrating their capacity to promote cancer invasion through direct contact with cancer cells.^41^ Schwann cells, known for their supportive role in nerve function, exhibit a contact‐dependent mechanism, prompting cancer cell migration toward nerves. Furthermore, the involvement of transforming growth factor beta (TGFβ) signaling in Schwann cells is implicated in fostering the aggressiveness of pancreatic ductal adenocarcinoma cells.^42^ Additionally, the expression of protocadherin 7 (PCDH7) in breast and lung cancer cells is noted, suggesting its participation in the assembly of carcinoma–astrocyte gap junctions, primarily composed of connexin 43.^43^ This intricate network of interactions underscores the need for further research to delineate the nuanced mechanisms through which nerves contribute to cancer progression, pinpointing specific nervous cell types and molecular pathways pivotal in this process.^44^

#### 3.1.2 Biological connection

The new technological interventions, particularly, nerve imaging and biological manipulation such as genetic engineering have also granted some progress in understanding the molecular mechanisms behind the interactions between cancers and nerves.^45^ The extracellular matrix, fibroblasts, adipose cells, immune‐inflammatory cells, blood, and lymphatic vascular networks are contents of the tumor microenvironment involved in tumor initiation, progression, and metastasis.^46^

When cancer cells develop from preneoplastic lesions to manifest cancer, nerve density nearly doubles compared with the non‐neoplastic tissue. Significantly, a study has shown that in breast cancer the nerve fibers correlated with poor differentiation, lymph node metastasis, triple‐negative subtype, and high clinical staging.^6^ Thus, all the above argument supports that nerves play crucial roles in tumor progression.

Biologically, there is a correlation between nerve fiber density and tumor size, margin status, lymph node metastasis, and pathological tumor, suggesting that the nerve is involved in angiogenesis related to tumor growth.^47^, ^48^ Besides, by the paracrine signaling mechanism chemically, the tumor cells are able to release neurotransmitters, neurotrophic factors, and axon guidance molecules to drive neuron reprogramming, and then recruit the nerves or invade the existing nerves. In addition, nerves also have the chemical ability to release neuroactive molecules to interact with the receptors of tumors or the tumor microenvironment for the metastasis cells to propagate, proliferate, and metastasize.^49^

### 3.2 Effect mechanisms

#### 3.2.1 Direct effect

**Figure 2:**
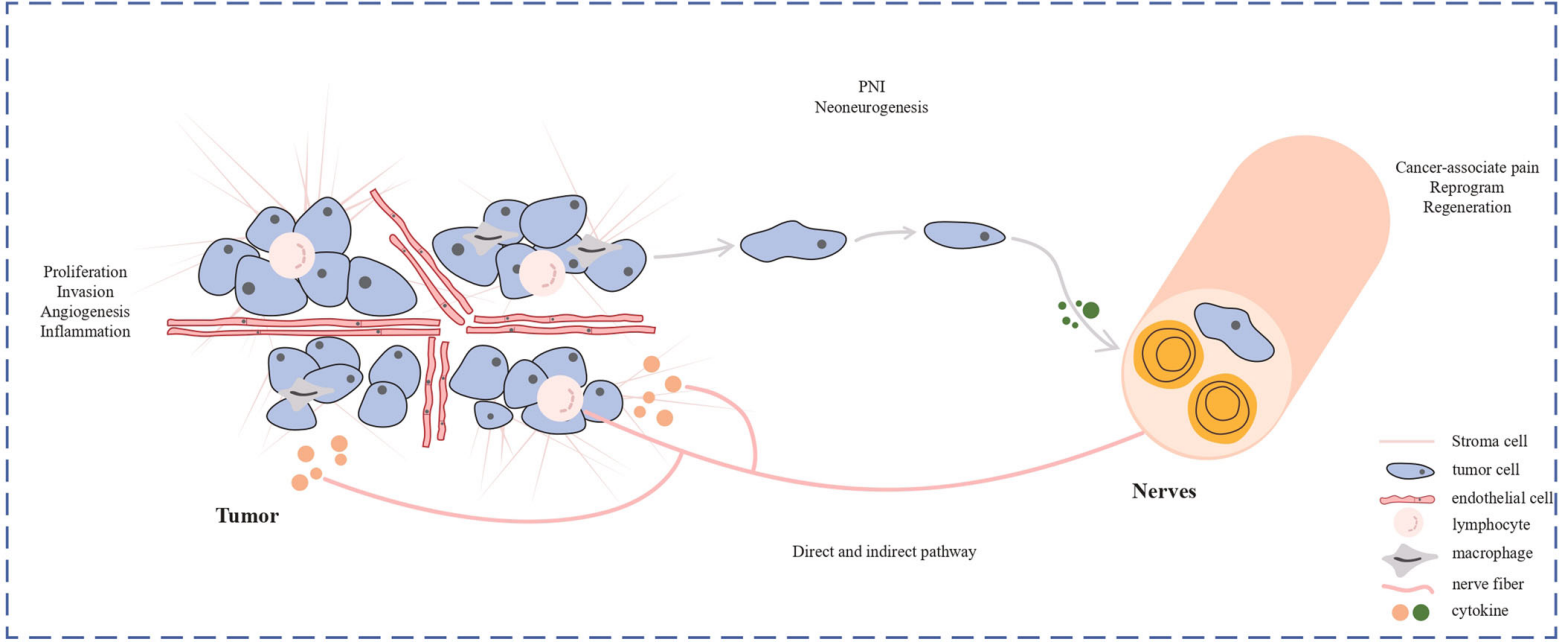
Nerve–tumor interaction/perineural invasion. Nerves can release neuroactive chemicals that stimulate tumor proliferation, invasion, angiogenesis, and inflammation by acting on tumor cells, lymphocytes, and macrophages. Tumor cells then travel to nerves and cause damage, and tumor cells also release cytokines, which promote neuronal reprogramming and regeneration. PNI, perineural invasion. [Color figure can be viewed at wileyonlinelibrary.com]

Cancer cells are transfused within or around nerve tissues in a process called the perineural invasion (PNI), which can be observed before the lymphatic or vascular invasion,^46^ and involves structural nerve damage that directly progresses to cancer‐associated pain, then the tumor cells invade neural tissue before the onset of tumorigenesis.^4^ Furthermore, at the early stage of cancer, the pancreas acinar cells (which constitute 90% of the pancreas epithelium) migrate along sensory neurons in the spinal cord, proving that PNI could be a potential route of metastasis (Figure 2).^50^

**Figure 3:**
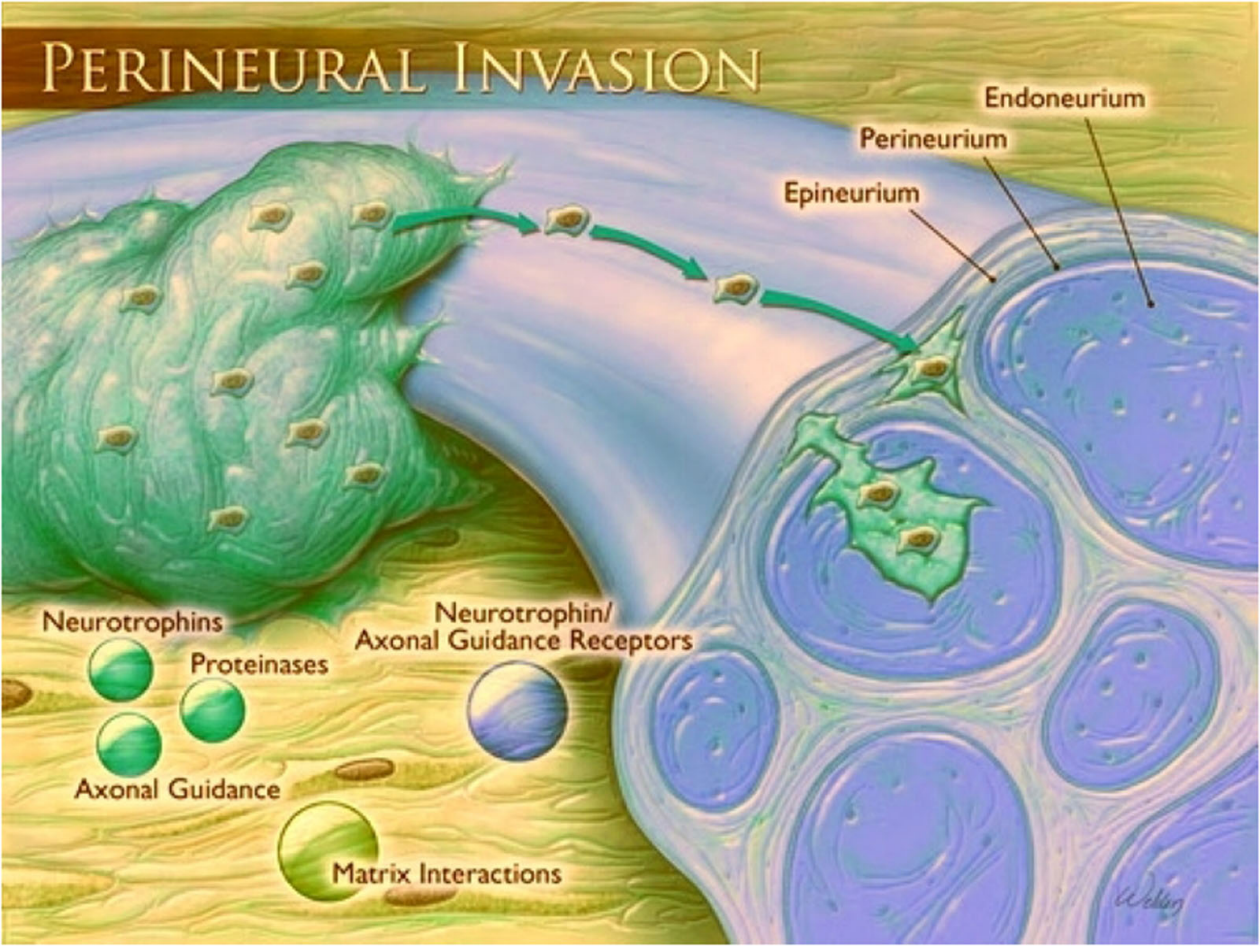
Perineural invasion and the structure of the peripheral nerve sheath. A tumor encircling a peripheral nerve is shown in cross‐section, indicating tumor cell spread. Tumor‐expressed molecules interact with stroma and receptors associated with peripheral neurons. [Color figure can be viewed at wileyonlinelibrary.com]

In an advanced stage, the process of perineural niche comes up invading cancer cells which leads to a cascade of inflammation cytokines caused by damage of the perineurium (Figure 3), and thence forming a unique cellular and biochemical microenvironment around the nerve. Cancer cells track along or around a nerve after infiltrating perineural space in the process of nerve injury, which in turn enhances neural regeneration.^51^ This niche includes diverse cellular components that may regulate the neural chasing to smoothen the PNI. Therefore, PNI is more commonly found in aggressive cancers, and the incidence rate of PNI was mentioned as up to 80% in head and neck cancers, 75% in prostate cancers, 98% in pancreatic cancers, 33% in CRCs, and 75% in cholangiocarcinoma.^52^, ^53^

The peripheral nerve sheath plays a crucial role in the PNI. Three connective tissue layers make up the nerve sheath. These layers are the epineurium, perineurium, and endoneurium, arranged from outside to inside (Figure 3).^54^ The epineurium, which connects one or more fascicles into a single nerve, is made up of two different layers: an inner layer made up of densely packed collagen fibrils and elastin fibers, and an exterior layer made up of areolar connective tissue and loosely distributed collagen bundles.^55^ The epineural component of the vasa nervorum, the extensive vascular network of the peripheral nerve, and the perineural lymphatic channels exist inside the areolar connective tissue on the outer section of the epineurium. Although this topic has historically been the subject of a lot of disputes in the PNI literature, the most current research indicates that these lymphatics do not enter the epineurium.^56^

An illustration of a tumor engulfing a peripheral nerve in cross‐section shows the spread of malignant cells (Figure 3). Tumor‐expressed molecules engage in interactions with the stroma around them and with receptors on peripheral neurons.^52^ The Schwann cells and distinct nerve axons are encased in the endoneurium or the deepest layer of the nerve sheath, which also forms a matrix surrounding individual nerve fibers. Endoneurial blood arteries have tight junctions as their endothelial lining instead of transendothelial channels. The blood–nerve barrier's most important characteristic is the relative impermeability of the endoneurial blood vessels, which is an extension of the perineurium's barrier function surrounding this compartment.^57^ PNI has been described as having tumor cells in every layer of the peripheral nerve sheath, ranging from small clusters of tumor cells inside a nerve that is surrounded by healthy tissue far from the tumor to well‐formed cancerous glands inside the perineurium or endoneurium.

After a review, we realized that few publications have claimed that tumor cells must be seen inside the perineurial layer,^52^, ^58^ but lately, the majority of research outcomes have argued that PNI results from the discovery of tumor cells in any of the three layers of the nerve sheath.^59^ Additionally, there is a lot of disagreement as to the extent of the difference in tumor–nerve contact required for PNI. When this happens outside the tumor's primary body, the result is more readily identified as a malignant carcinomatous invasion of a neurological structure. However, it is less obvious when this takes place within the tumor's primary body.

To be proved as PNI, at least 33% of the nerve's circumference must be encircled by tumor cells; anything less than this percentage will be a localized abutment rather than an invasion.^60^

**Figure 4:**
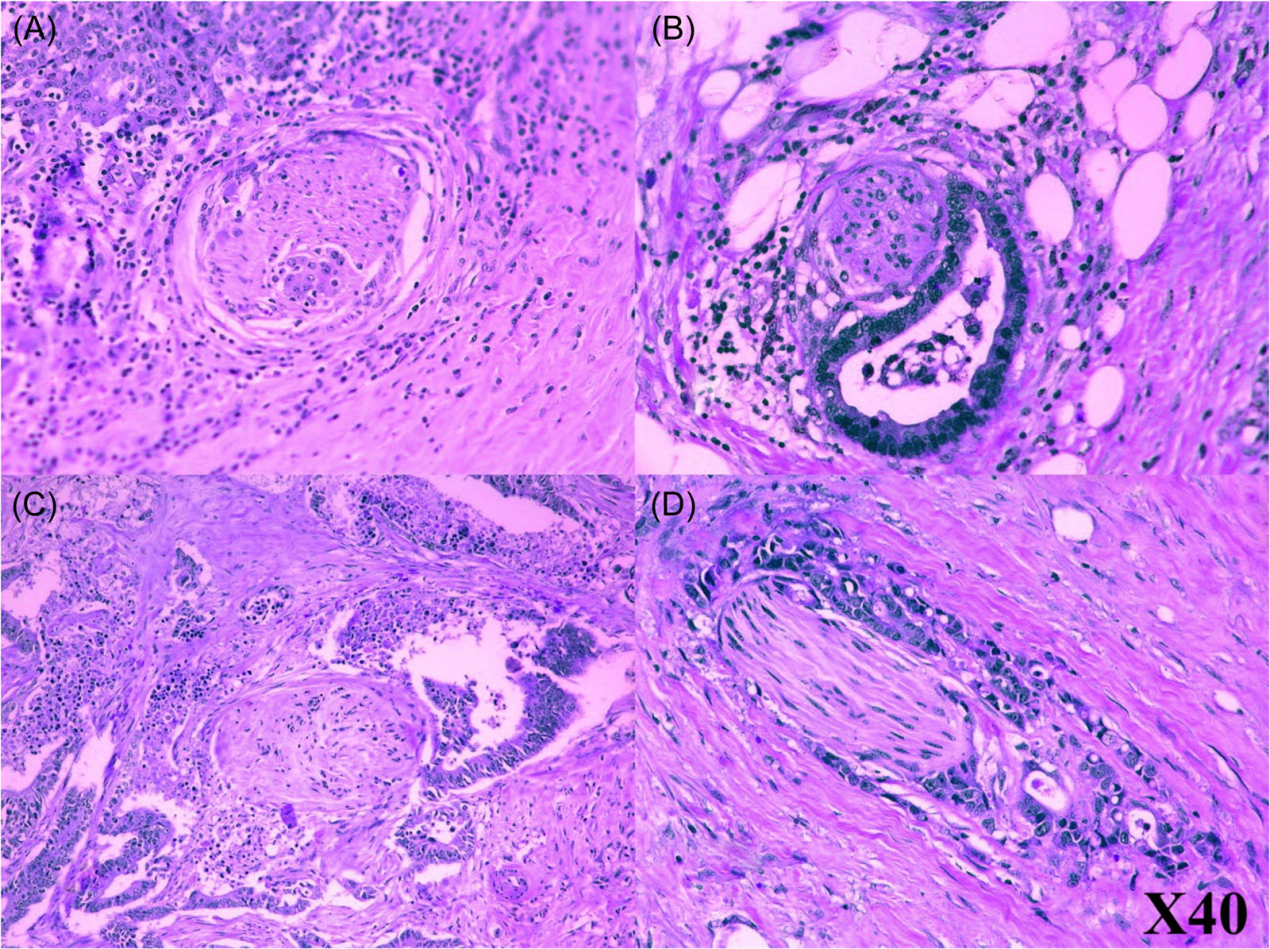
Perineural invasion. Photomicrographs were employed to depict instances of perineural invasion in specimens of human colorectal cancer subjected to hematoxylin and eosin staining. It shows that tumor cells develop either in clusters (A) or glandular elements (B), and the nerve is within the core body of the tumor (C) or outside the tumor (D). [Color figure can be viewed at wileyonlinelibrary.com]

Hematoxylin and eosin‐stained sections of human colorectal tumors were examined for PNI by a pathologist (Figure 4). PNI is present when tumor cells develop either in clusters (Figure 4A) or glandular elements (Figure 4B) within/inside the peripheral nerve sheath. To diagnose PNI, at least 33% of the circumference of the nerves must be affected by tumor cells, even if tumor cells are not inside the nerve sheath but are near the perineural environment. As shown in Figure 4, the nerve is within the core body of the tumor or outside the tumor (Figure 4C,D).^52^

**Figure 5:**
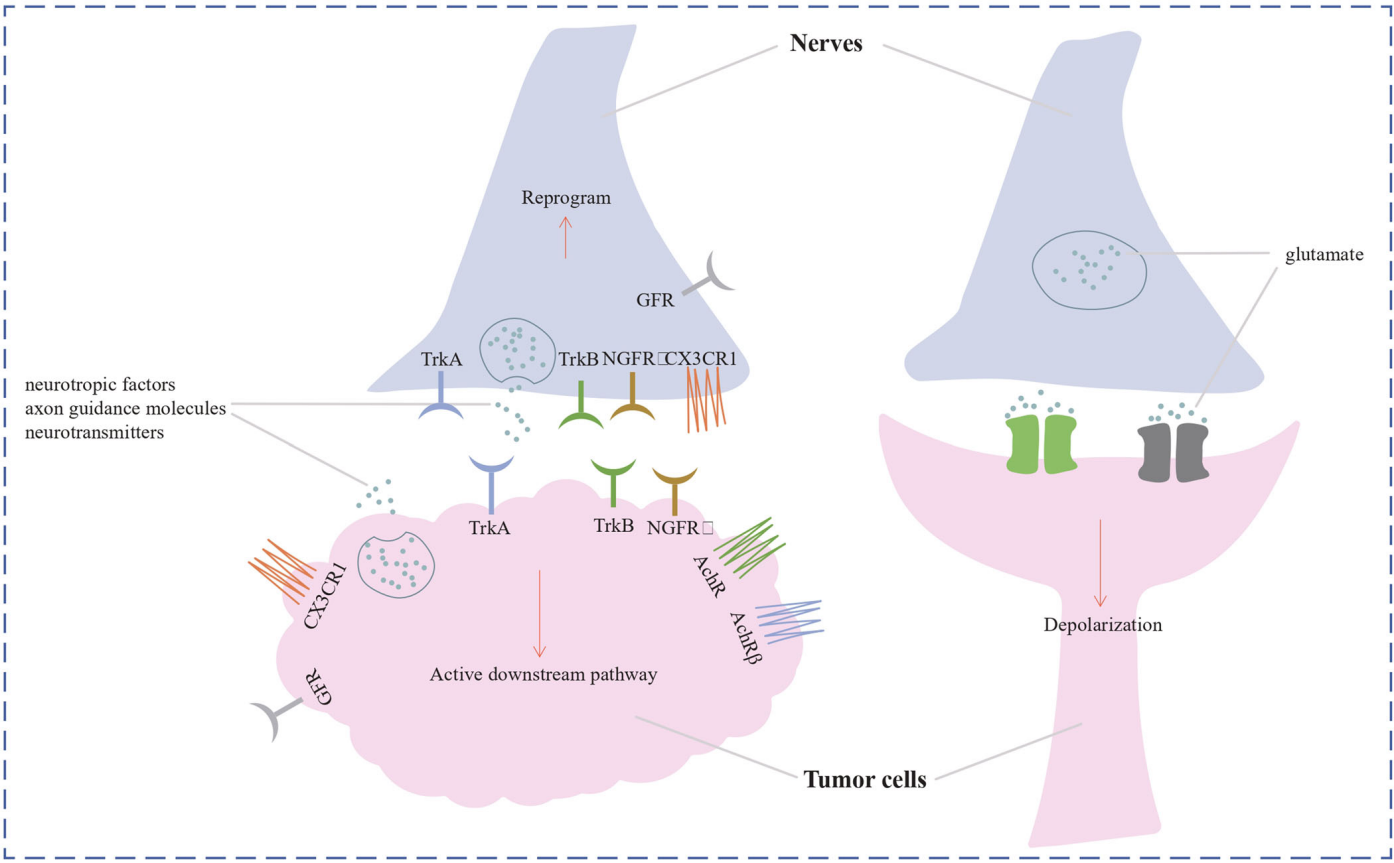
Nerve–tumor interaction/paracrine mode. Tumors release molecules that are also secreted by nerves, such as neurotropic factors, axon guidance molecules, and neurotransmitters. These bioactive compounds interact with nerve receptors, influencing the reprogramming of nerves, while concurrently acting on tumor receptors to initiate downstream signaling. Beyond the conventional paracrine model, a noteworthy complexity emerges, as tumors and nerves establish synaptic connections, facilitating depolarization processes that actively contribute to the promotion of tumor progression. This intricate interplay underscores the multifaceted nature of the communication between tumors and nerves, extending beyond conventional signaling mechanisms to encompass synaptic interactions that significantly impact the dynamic evolution of the tumor microenvironment. [Color figure can be viewed at wileyonlinelibrary.com]

Thus, nerve activity is impacted by tumors, and nerves also play a crucial role in tumor growth via either direct or indirect channels. Because tumor cell membranes have receptors that react to neurotropic substances or neurotransmitters, the nerves release these substances in a paracrine way to accelerate tumor growth. To deliver the excitatory signal, innervation might release neurotransmitters straight into a synapse created by neurons and tumor cells (Figure 5).

Through the paracrine mechanism, nerves, particularly neurons and Schwann cells, can modify the biological activity of cancer cells and affect the development of tumors. From a general view, the three main groups of neuroactive chemicals generated by the nerves involved in tumor–nerve interaction are as follows: (a) neurotropic substance including brain‐derived neurotrophic factor (BDNF), glial cell line‐derived neurotrophic factor (GDNF), and nerve growth factor (NGF); (b) axon guidance molecules, such as C‐X3‐C motif chemokine ligand 1 (CX3CL1), EPH receptor A2 (EPHA2), C‐C motif chemokine ligand 2 (CCL2), Slit, and so forth; and (c) neurotransmitters, such as glutamate, acetylcholine (ACh), epinephrine, glycine, dopamine, norepinephrine, and so forth.^61^ Chemically, tumor cells express a variety of receptors when in response to numerous neuroactive substances to activate downstream pathways, including tyrosine kinase receptor A (TrkA), tyrosine kinase receptor B (TrkB), and NGF receptor (NGFR). The neuroactive chemicals and receptors linked to tumor growth have been known for decades. Figure 5 also depicts this mode of communication between tumor cells and neurons.^62^, ^63^

The chemical synapse, a structure that typically comprises two nearby neurons interacting through neurotransmitters like glutamate, is a unique instance of crosstalk between cancers and nerves.^49^ Thus, in gliomas, electron microscopy also revealed synaptic structures including presynaptic neurons and postsynaptic tumor cells by capturing glioma cell excitatory postsynaptic potentials.^64^ Similar to synapses produced between neurons, neurogliomal synapses could operate. The postsynaptic area of glioma cells had a high prevalence of aminomethylphosphonic acid receptors, according to gene expression analyses and confocal imaging.^65^ Additional research on this receptor revealed that it was responsible for a glioma cell's depolarization, which propagated to other glioma cells in the network by way of their gap junctions. Importantly, although inhibiting depolarization brought on by synaptic activity, neuronal activity or depolarization might encourage tumor growth and spread.^66^

#### 3.2.2 Indirect effect

##### 3.2.2.1 Angiogenesis

Previously mentioned studies have demonstrated that nerves directly control stromal tissues.^67^ Conversely, nerves also interact with a variety of stromal elements in the tumor microenvironment by indirectly encouraging tumor development and spread. Angiogenesis, the process in which new capillary vessels are created from the existing vasculature, makes the endothelial cells activate, multiply, and migrate, and this is essential for the development and spread of tumors.^68^, ^69^ Angiogenesis enables tumors to grow by developing/producing their own nutrition and oxygen, thus, it is related to tumor outcomes and reveals how aggressive tumor cells are.^45^

The nerves generate neurotrophic factors and transmitters that attach to receptors and cause endothelial cells to migrate, which contribute to the process of angiogenesis. Zahalka et al.^70^ showed that by changing the metabolism of blood vessel endothelial cells, adrenergic nerves controlled the angiogenesis of microenvironment in the prostate cancer. *ADRB2* gene prevented/inhibited the endothelial oxidative phosphorylation that produced angiogenesis. Through angiogenesis, the metabolic change brought on by nerves facilitated the development of prostate tumors.^70^ Finally, neoneurogenesis and angiogenesis certainly have some parallels, and both have the same receptors and are controlled by comparable transmitters and neurotrophic substances.^71^

Tumor cells may draw neural progenitors throughout the process of tumor development, which triggers neurogenesis to enable their proliferation and dissemination. Adrenergic nerve fibers and recently established neural networks grow and infiltrate into the stroma associated with cancer, supplying signals to control tumor development.^72^ The involvement of adrenergic nerve fibers in the angiogenic switch, which stimulates endothelial cells to facilitate exponential tumor development, has been studied before concerning prostate cancer. This work demonstrates this role.^70^

##### 3.2.2.2 Immunity

**Figure 6:**
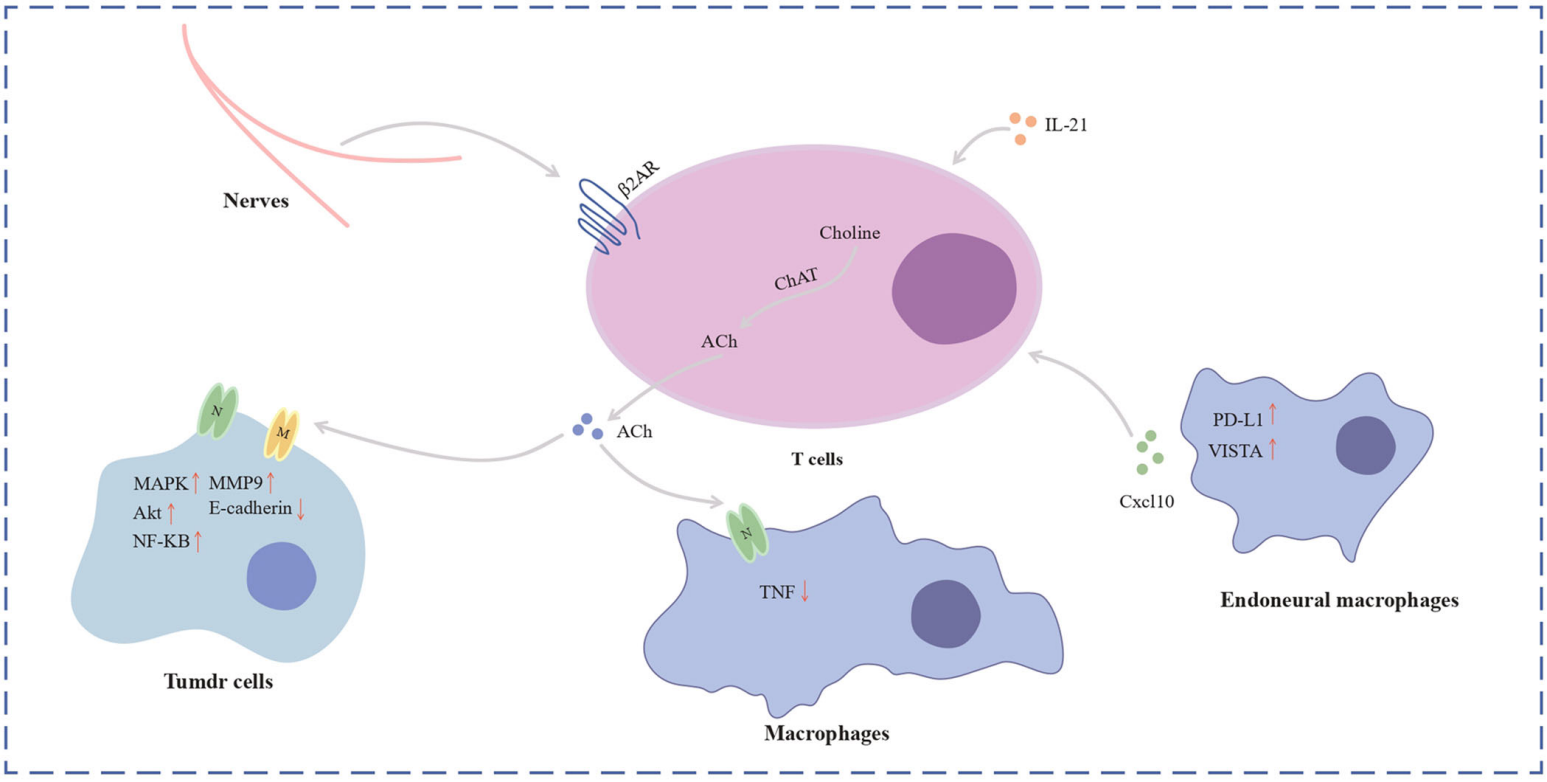
Nerves–immune cells interaction. The enzyme choline acetyltransferase produces acetylcholine (ACh) in T cells. Adrenergic innervation enhances the generation of T‐cell‐derived ACh, which is implicated in tumor immunology and tumor biological behavior regulation. Endoneural macrophages are also involved in tumor metastasis regulation. [Color figure can be viewed at wileyonlinelibrary.com]

Nerves are part of the tumor microenvironment and interact with the immune system, which may speed up the development of the tumor via inflammation, and the majority of the molecular signals and receptors that regulate neuroendocrine and neural pathways as well as immunological responses belong to the same superfamily.^45^, ^73^ The adrenergic innervation, for instance, was discovered to enhance ACh synthesis of the β2‐adrenergic receptor (β2‐AR) that expresses T cells in the spleen (Figure 6).^74^ According to recent reports, ACh is crucial for controlling immunity, particularly immunity to cancer. Through the α7 nicotinic, ACh receptor produced by cytokine‐producing macrophages, T‐cell‐derived ACh can inhibit the generation of tumor necrosis factor (TNF).^75^ In addition, the proliferation, migration, and invasion of lung cancer are sped up by the released ACh, which binds back to nicotinic and muscarinic receptors on the cells.^76^ In fact, choline acetyltransferase, which catalyzes the production of ACh from choline in both CD4+ and CD8+ T cells, is substantially activated by IL‐21 to control T‐cell migratory and immunological activities.^77^ These studies concluded that the autonomic nervous system can directly control the immune system, to indirectly involve the development and progression of tumors.

The crucial steps in preventing tumor growth and development are tumor lymphocyte infiltration and activation.^78^ However, by activating immunological checkpoint pathways that block antitumor immune responses, tumor cells are able to evade immunosurveillance.^79^ According to a retrospective analysis of patients with breast cancer, immune checkpoint molecule expression and clinical outcomes were linked with sympathetic and parasympathetic nerve density, which developed an explanation that in animal samples breast cancer immune checkpoint molecule expression was decreased by genetic parasympathetic neurostimulation and sympathetic nerve denervation.^80^

These findings suggested a strong relationship between nerves and immunological checkpoint treatment and partially explained the contradictory effects of sympathetic and parasympathetic nerves in breast cancer. However, further research is needed to clarify the mechanism through which neurons control immunological checkpoint molecules.^81^

##### 3.2.2.3 Chemical impact of neurotransmitter and neurotrophic factors on tumor growth

A crucial component of PNI could involve axonal migration. Axonal guiding molecules and neurotrophic growth factors are both necessary for the intricate process of axonal development. The family of neurotrophic factors known as neurotrophins, which also includes NGF, BDNF, neurotrophin 3 (NT‐3), and neurotrophin 4/5 (NT‐4/5), are best defined.^82^ The neurotrophins are excellent research subjects for the PNI pathway because of their strong effects on neuronal development. A rising corpus of research links these molecules to cancer, along with other neurotrophic factors. Table 2 describes the potential roles and cancer‐related expression patterns of neurotrophic factors.

**Table 2:** Neurotrophic factors—Possible functions and expression patterns in cancer.

Factor	Expression in cancer	Function in cancer
NGF	Through its interaction with TrkA, an NGF‐specific receptor may promote the proliferation of epithelial cancer cells and facilitate nerve invasion; binding of NGF to TrkA results in the activation of the p44/42 MAPK signaling cascade and upregulation of MMP‐2, a proinvasive mediator.	Strongly expressed in the perineurium of peripheral nerves and overexpressed in the cell lines of prostate and pancreatic cancer.^83^
BDNF	Promotes neurite development and may be overexpressed by tumor cells; increases tumor cell invasion at low‐to‐moderate doses.	Overexpressed in adenoid cystic carcinoma and pancreatic cancer; expression does not correlate with PNI, indicating that the BDRM‐expressing phenotype may develop before nerves.^84^
GDNF	Affects tumor cells in a chemotactic and chemokinetic manner and causes elevated MMP‐9 production and activity.	The RET protein tyrosine kinase receptor for GDNF is overexpressed in human neuroplexi tissues and is present in several pancreatic cancer cell lines.^85^
NT‐3	At low‐to‐moderate doses, it stimulates tumor cell invasion.	Overexpressed in samples of pancreatic cancer.^86^

Abbreviations: BDNF, brain‐derived neurotrophic factor; GDNF, glial cell line‐derived neurotrophic factor; MAPK, mitogen‐activated protein kinases; MMP‐2, matrix metalloproteinase‐2; MMP‐9, matrix metalloproteinase‐9; NGF, nerve growth factor; NT‐3, neurotrophin 3; PNI, perineural invasion; TrkA, tyrosine kinase receptor A.

By crossing all layers of the nerve sheath, cancer cells invade the nerve. Once the cancer cells have entered the nerve, they have found a suitable environment to spread intraneurotically and make progress for the disease. Cancer cell invasion has been characterized as cancer cells present in one of the layers of the nerve sheath or as cancer cells present in the endoneurium connected with the Schwann cells.^87^, ^88^ Contrary to instances with PNI but no intraneural invasion, the intraneural invasion has been linked to a greater probability of local/distant recurrence in pancreatic ductal adenocarcinoma (PDAC).^47^ Cancer‐related neurogenesis is proven in prostate cancer as a novel biological phenomenon because prostate lesions that are both paraneoplastic and neoplastic have a higher density of nerve fibers.^6^

Furthermore, PNI would have a fertile ground when cancer cells directly attach to nerves. Additionally, a variety of neurotransmitters, NPs, and signal transduction pathways control the pathophysiology of cancer cells.^89^, ^90^ Cancer cells can interact with neurotransmitters and release neurotransmitters in return, which can activate receptors on nerve fibers. This stimulation may result in nerve fiber network dysregulation and PNI acceleration.

One of the methods for cancer treatments is chemotherapy which depends on preventing/minimizing the migration of tumor cells and its invasion, by inhibiting the neurotransmitters and their receptors,^91^ of which norepinephrine is an indispensable neurotransmitter that is secreted by the sympathetic postganglionic neurons, and is active in cell migration. Given that the formation of cancer metastases requires the migration of malignant cells, norepinephrine acts on β‐2 adrenergic receptors to actively mediate the malignant migration, and β‐blockers can affect this action.^92^ According to earlier research on mice, neurotrophin production is enhanced when norepinephrine is stimulated throughout the development. Gemcitabine, a medication frequently used to treat pancreatic cancer, worked better when neurotrophin receptors were inhibited.^93^ A research showed that a differentiation signature implicated in the process as mentioned earlier, and cancer development drastically downregulated β‐2 and α‐2 adrenergic receptors.

ACh is another neurotransmitter that plays a critical role in metastasis progression by the cholinergic system and the nicotinic acetylcholine receptors that bind to it operate as neurotransmitters and promote the development of PDAC and cholangiocarcinoma (CCA). More lymph node metastases and larger concentrations of muscarinic ACh receptor 3 (M3) were associated with high‐grade differentiation of PDAC,^94^ similarly, the proliferation and expansion of CCA cells are also influenced by the cholinergic system.

Numerous studies on NGF have revealed that NGF directly affects not only the peripheral PNS and CNS but also the elements of the tumor microenvironment. Besides, by encouraging cancer cells to maintain a more differentiated cell phenotype, NGF therapy can slow the development of tumors.^95^ Likewise, due to its capacity to influence immune cell functions, NGF is able to play a significant part in the defence against cancer. Tropomyosin receptor kinase A (TRKA) and the p75 neurotrophin receptor are two significant cell surface receptors for neurotrophins.^96^, ^97^ A study showed that the proliferation and invasiveness of malignancy cells that are stimulated by NGF are marked by high levels of NGF and high levels of TRKA.^98^ In this model, it seems that anti‐NGF therapy only seemed to slow down neural inflammation, invasion, and metastasis when the disease had just recently started. This suggests that it would be crucial to time the start of therapy.

## 4 DISCUSSION

Interactions between tumors and the nervous system have arisen as a prominent feature in human cancer. Within the CNS, intricate dialogues unfold between neurons and tumor cells, thereby facilitating the proliferation of glioma and neuroblastoma. Additionally, the PNS assumes a critical role in the reconfiguration of the tumor microenvironment, exerting influence over angiogenesis and immune cell functionality. Concurrently, it plays a direct role in fostering both tumorigenesis and metastasis. In a bidirectional relationship, cancer cells in turn exert influence on neuronal function, heightening neuronal activity and modulating the functionality of the circuits into which cancer cells integrate both structurally and electrically. Cancer cells are transfused within or around nerve tissues in a PNI mechanism, which can be observed before the lymphatic or vascular invasion, and involves structural nerve damage that directly progresses to cancer‐associated pain, then the tumor cells invade neural tissue before the onset of tumorigenesis as nerves directly control stromal tissues.

This review indicated that the activity of glutamatergic neurons within the CNS promotes the proliferation of glial precursor cells and the growth of malignant gliomas. This occurs through the concerted activity of glutamatergic neurons and cancer cells, establishing integration with the brain's neural network. Consequently, cancer cells receive impulses that seemingly contribute to the stimulation of tumor growth. On the other hand, indirect effects like the paracrine mechanism particularly the neurons and Schwann cells can modify the biological activity of cancer cells and affect the development of tumors. Also, BDNF, GDNF, NGF, and axon guidance molecules, such as CX3CL1, EphA2, CCL2, Slit, and so forth, are groups of neuroactive chemicals generated by the nerves involved in tumor–nerve interaction.

This study summarizes the key findings and main points of the most up‐to‐date 95 previous studies of the oncology field which belongs to the neuroscience of cancer as a narrative review. Table 3 demonstrates the main literature review and its most significant outcomes that serve our study as an emerging neuroscience of cancer.

**Table 3:** Summary of the main literature review.

Title	References	Key points of finding
	Cancer–neuronal connection	
A role for hypocretin/orexin in metabolic and sleep abnormalities in a mouse model of nonmetastatic breast cancer.	Borniger et al.^29^	Neuron–cancer interaction and functional effect could be surmised by the influence of nervous system elements.
Neuronal activity promotes glioma growth through neuroligin‐3 secretion.	Venkatesh et al.^31^	The growth of malignant gliomas is driven by the activity of glutamatergic neurons.
PIK3CA variants selectively initiate brain hyperactivity during gliomagenesis.	Yu et al.^33^	Released signals from brain tumors “gliomas” impact the function of invaded neural circuits by encouraging aberrant synaptogenesis.
Progenitors from the central nervous system drive neurogenesis in cancer.	Mauffrey et al.^35^	Malignancy cells invade the peripheral nerve tissue by a mechanism called “per neural invasion.”
Nerve growth factor is a potential therapeutic target in breast cancer.	Adriaenssens et al.^3^	Nerve growth factor (NGF), the prototypic neurotrophin, could be targeted in breast cancer to inhibit tumor cell proliferation, survival, and metastasis.
	Chemical connection	
Neuropeptide Y and its role in CNS disease and repair.	Decressac et al.^36^	Neuropeptide Y is secreted from tumor cells and acts through the Y2 receptor (Y2R) to mediate proliferation and angiogenesis during cancer development.
Beta‐blockers and breast cancer mortality: a population‐based study.	Barron et al.^40^	Inhibiting the β2‐adrenergic signaling pathway, potentially through the use of beta‐blockers will exert a mitigating effect on breast cancer progression and mortality.
Early pancreatic cancer lesions suppress pain through CXCL12‐mediated chemoattraction of Schwann cells.	Demir et al.^42^	Involvement of (TGFβ) signaling in Schwann cells is implicated in fostering the aggressiveness of pancreatic ductal adenocarcinoma cells.
Schwann cells support the oncogenic potential of pancreatic cancer cells through TGFβ signaling.	Roger et al.^43^	Protocadherin 7 (PCDH7) in breast and lung cancer cells and its participation is noted in the assembly of carcinoma–astrocyte gap junctions, primarily composed of connexin 43.
	Further studies suggested to delineate the chemical mechanism pathways in cancer–nerve correlation.	
	Biological connection	
New horizons in tumor microenvironment biology: challenges and opportunities.	Chen et al.^46^	Extracellular matrix, fibroblasts, adipose cells, immune‐inflammatory cells, blood, and lymphatic vascular networks, all are contents of the tumor microenvironment which belong to tumor initiation, progression, and metastasis.
Nerve fibers in the tumor microenvironment in neurotropic cancer, pancreatic cancer, and cholangiocarcinoma.	Tan et al.^91^	Correlation between nerve fiber density and tumor size, margin status, lymph node metastasis, and pathological tumor.
	Effect mechanisms—Direct effect	
The dynamic synapse.	Choquet et al.^49^	Cancer cells are transfused within or around nerve tissues in a process called the perineural invasion “PNI”
Syndecan‐2 promotes perineural invasion and cooperates with K‐ras to induce an invasive pancreatic cancer cell phenotype.	De Oliveira et al.^50^	Early stage of cancer the pancreas acinar cells migrate along sensory neurons in the spinal cord, proving that PNI could be a potential route of metastasis.
Treatment and prognosis for patients with intrahepatic cholangiocarcinoma: systematic review and meta‐analysis.	Mavros et al.^53^	In an advanced stage, the process of perineural niche comes up invading cancer cells which leads to a cascade of inflammation cytokines caused by damage of the perineurium.
Cellular and molecular mechanisms of perineural invasion of pancreatic ductal adenocarcinoma.	Li et al.^57^	PNI has been described as having tumor cells in every layer of the peripheral nerve sheath, ranging from small clusters of tumor cells inside a nerve that is surrounded by healthy tissue far from the tumor to well‐formed cancerous glands inside the perineurium or endoneurium.
Perineural invasion‐associated biomarkers for tumor development.	Liu et al.^56^	At least 33% of the nerve's circumference must be encircled by tumor cells.
Neurotransmitters: emerging targets in cancer.	Jiang et al.^63^	Through the paracrine mechanism, nerves, particularly neurons and Schwann cells, can modify the biological activity of cancer cells and affect the development of tumors.
	Effect mechanisms—Indirect effect Angiogenesis	
Angiogenesis in pancreatic cancer: current research status and clinical implications*.*	Li et al.^69^	Angiogenesis, a process in which new capillary vessels are created by existing vasculature, makes the endothelial cells activate, multiply, and migrate, which is essential for the development and spread of tumors.
Role of the nervous system in cancers: a review.	Wang et al.^45^	Angiogenesis enables tumors to grow by developing/producing their own nutrition and oxygen and reveals tumor aggressiveness.
Tumor angiogenesis: causes, consequences, challenges, and opportunities.	Lugano et al.^71^	Neoneurogenesis and angiogenesis certainly have some parallels, and both have the same receptors and are controlled by comparable transmitters and neurotrophic substances. And through angiogenesis, the metabolic change brought on by nerves facilitated the development of prostate tumors.
	Effect mechanisms—Indirect effect Immunity	
Neural regulations of the tumor microenvironment.	Lugano et al.^73^	Nerves and the immune system are a part of the tumor microenvironment, which may speed up the development of the tumor via inflammation, and the majority of the molecular signals and receptors that regulate neuroendocrine and neural pathways.
α7‐ and α9‐Containing nicotinic acetylcholine receptors in the functioning of the immune system and in pain.	Shelukhina et al.^75^	Adrenergic innervation, for instance, was discovered to enhance ACh synthesis, the β2‐adrenergic receptor (β2‐AR) that expresses T cells in the spleen, ACh is crucial for controlling immunity, particularly immunity to cancer.
Acetylcholine signaling system in the progression of lung cancers.	Friedman et al.^76^	Lung cancer cells' proliferation, migration, and invasion are sped up by the released ACh, which binds back to nicotinic and muscarinic receptors on the cells.
Immune checkpoint inhibitors: recent progress and potential biomarkers.	Darvin et al.^79^	Activating immunological checkpoint pathways that block antitumor immune responses, tumor cells can evade immunosurveillance.
Genetic manipulation of autonomic nerve fiber innervation and activity and its effect on breast cancer progression.	Kamiya et al.^80^	In breast cancer immune checkpoint molecule expression was decreased by genetic parasympathetic neurostimulation and sympathetic nerve denervation.
	Effect mechanisms—Indirect effect Neurotransmitter's chemical impact	
A comprehensive analysis of neurotrophins and neurotrophin tyrosine kinase receptors expression during development of zebrafish.	Nittoli et al.^82^	The neurotrophins (NGF, BDNF, NT‐3, NT‐4/5) are excellent research subjects for the PNI invasion pathway because of their strong effects on neuronal development.
Limitations of nerve fiber density as a prognostic marker in predicting oncological outcomes in hepatocellular carcinoma.	Bednarsch et al.^90^	A variety of neurotransmitters, neuropeptides, and signal transduction pathways control the pathophysiology of cancer cells.
Nerve fibers in the tumor microenvironment in neurotropic cancer, pancreatic cancer, and cholangiocarcinoma.	Tan et al.^91^	Chemotherapy depends on preventing/minimizing the migration of tumor cells and their invasion, by inhibiting the neurotransmitters and their receptors.
β2 adrenergic‐neurotrophin feedforward loop promotes pancreatic cancer.	Renz et al.^93^	Norepinephrine acts on β‐2 adrenergic receptors to actively mediate the malignant migration, and β‐blockers can affect this action.
High expression of muscarinic acetylcholine receptor 3 predicts poor prognosis in patients with pancreatic ductal adenocarcinoma.	Zhang et al.^94^	Acetylcholine (Ach) plays a critical role in metastasis progression by the cholinergic system and the nicotinic acetylcholine receptors to promote the development of pancreatic ductal adenocarcinoma.
High‐affinity TrkA and p75 neurotrophin receptor complexes: A twisted affair.	Conroy et al.^97^	NGF can play a significant part in the defense against cancer.
Loss of p53 drives neuron reprogramming in head and neck cancer.	Amit et al.^72^	Adrenergic nerve fibers and recently established neural networks grow and infiltrate into the stroma associated with cancer, supplying signals to control tumor development.

In this study, we present a novel approach to carefully describe the cancer invasion in the nervous system, that addresses the limitations of existing understanding and visualization. Our innovation involves the interactions between neurology and cancer are multifaceted, bearing various clinical ramifications that could potentially advance neuroscience‐driven cancer therapies and refine treatments for neurological disorders and cancer‐associated pain, which utilizes a pivotal efficiency to develop biomarkers and pinpoint the most promising therapeutic targets in this context. Unlike previous approaches that focus on one side or aspect, our method offers an all‐out coverage of the aspects of tumor invasion on neuronal cells and nerves, accordingly improving the accuracy or efficiency of a good understanding of this field. Furthermore, this innovation opens up opportunities for further research and applications of therapy strategies especially those related to controlling neurotransmitters, neurotrophic factors, and immunological checkpoint pathways. Overall, our work contributes to the broader significance of the oncology field by providing a new and impactful description of the cancer invasion in the nervous system and beneficial reference for direct, indirect, biological, and chemical interaction between cancer invasion and the nervous system.

## 5 CONCLUSIONS

The review highlights the significant influence of neurons on tumor growth and progression, emphasizing the importance of neuronal involvement in oncology. It provides a detailed examination of the mechanisms through which tumors invade neuronal cells and nerves, including biological and chemical interactions that contribute to cancer development. Additionally, the review explores how manipulating neurotransmitters can positively affect cancer growth, suggesting new therapeutic strategies that target these pathways to control tumor progression.

The future perspective of neuroscience‐informed cancer therapies relies heavily on the identification of viable biomarkers for nervous system–cancer interactions, alongside the capacity to conduct clinically meaningful trials, as we found that there is a lack of clinical trials related to nerve tissues healing after cancer invasion and how to prolong the nerves life. Besides, further studies should be conducted to understand more about the relationship between neurons and cancer cells, and therefore to establish new therapy techniques and mechanisms by controlling and modifying neuron networks that supply signals to tumors.

## AUTHOR CONTRIBUTIONS

Issam AbuQeis is wrote and edited the original manuscript. Abeer A. Teeti contributed to collecting literature and editing English writing. Yu Zou and Ying‐Chun Ba downloaded the literature, supervised, and revised the manuscript.

## CONFLICT OF INTEREST STATEMENT

The authors declare no conflict of interest.

## ETHICS STATEMENT

Not applicable.

## TRANSPARENCY STATEMENT

All information added in this study is according to scientific research guidelines, with full transparency of citation.

## Data Availability

Data reported in this study are available from the corresponding author upon reasonable request.
